# Impact of Silicon in Plant Biomass Production: Focus on Bast Fibres, Hypotheses, and Perspectives

**DOI:** 10.3390/plants6030037

**Published:** 2017-09-09

**Authors:** Marie Luyckx, Jean-Francois Hausman, Stanley Lutts, Gea Guerriero

**Affiliations:** 1 Groupe de Recherche en Physiologie Végétale (GRPV), Earth and Life Institute—Agronomy (ELI-A), Université catholique de Louvain (UCL), B-1348 Louvain-la-Neuve, Belgium; marie.luyckx@uclouvain.be; 2 Environmental Research and Innovation Department, Luxembourg Institute of Science and Technology, L-4362 Esch/Alzette, Luxembourg; jean-francois.hausman@list.lu

**Keywords:** fibre crops, silicon, bast fibres, phytohormones, intrusive growth, lignocellulosic biomass

## Abstract

Silicon (Si) is an abundant element which, when supplied to plants, confers increased vigor and resistance to exogenous stresses, as well as enhanced stem mechanical strength. Plant species vary in their ability to take Si up and to accumulate it under the form of silicon dioxide (SiO_2_) in their tissues: emblematic of this is the example of Poales, among which there is rice, a high Si accumulator. Monocots usually accumulate more Si than dicots; however, the impact that Si has on dicots, notably on economically important dicots, is a subject requiring further study and scientific efforts. In this review, we discuss the impact that Si has on bast fibre-producing plants, because of the potential importance that this element has in sustainable agriculture practices and in light of the great economic value of fibre crops in fostering a bio-economy. We discuss the data already available in the literature, as well as our own research on textile hemp. In particular, we demonstrate the beneficial effect of Si under heavy metal stress, by showing an increase in the leaf fresh weight under growth on Cd 20 µM. Additionally, we propose an effect of Si on bast fibre growth, by suggesting an action on the endogenous phytohormone levels and a mechanical role involved in the resistance to the turgor pressure during elongation. We conclude our survey with a description of the industrial and agricultural uses of Si-enriched plant biomass, where woody fibres are included in the survey.

## 1. Introduction

Si is the second most abundant element in soils and can be found in noticeable concentration in many terrestrial plants [1,2]. Plant species vary in their ability to take up and accumulate Si as silicon dioxide (SiO_2_) in their tissues. Depending on this characteristic, plants are classified as excluders, intermediate types, or accumulators [3,4]. Most dicots accumulate less than 0.1% Si on a dry weight basis, but many grass species are able to accumulate as much as 10% [4,5], and rice is often considered as the most effective Si-accumulating plant [6]. To date, Si has not been recognised as an essential element, although numerous studies have clearly demonstrated that Si is beneficial for plant growth and development, especially under a wide range of (a)biotic stress conditions [7,8,9,10].

Si deposition occurs mainly as phytoliths (SiO_2_·*n*H_2_O) [7,11]. It acts as a physical barrier and thus improves plant resistance to pathogens and insects, by increasing the rigidity and abrasiveness of plant tissues, thereby reducing their digestibility to herbivores [11,12].

Najihah and colleagues observed that the accumulation of Si in epidermal and endodermal cell walls protected oil palm roots from the penetration of the fungus *Ganoderma boninense* [13]. Si depositions also protect plants against lodging through stem strengthening [14]. Si improves drought resistance by thickening leaves, which is beneficial to reduce the transpirational loss of water [15], or by increasing cell wall stability in stressed plants [16].

Si remaining in the form of soluble silicic acid (Si(OH)_4_) may be involved in biochemical/molecular processes contributing to growth stimulation and stress resistance in relation to the oversynthesis of stress hormones such as salicylic acid (SA), jasmonic acid (JA), and ethylene [10,17]. Si alleviation of damages caused by toxic ions (salt and heavy metal stress) includes, among others, the regulation of genes involved in their uptake and translocation, changes in their symplastic and apoplastic distribution, improvement of absorbed light allocation, stimulation of antioxidant enzymatic activities (SOD, POD, CAT, GR), and non-enzymatic antioxidant synthesis [8,14,16,18,19,20,21].

The cell wall plays a key role in plant response to environmental cues, but it also constitutes a specific target for the deposition of silica bodies; silicification is indeed frequently associated with cell wall thickening [22]. Accordingly, there is an increasing interest in deciphering the underlying processes leading to cell wall structural modification and in furthering our understanding of the impact of Si on some plants, such as fibre crops, which are cultivated for their biomass.

## 2. Impact of Si on Bast Fibre-Producing Plants: Hemp as an Example

Bast fibres are produced by fibre crops such as flax (*Linum usitatissimum* L.), hemp (*Cannabis sativa* L.), ramie (*Boehmeria nivea* L. Gaud.), nettle (*Urtica dioica* L.), jute (*Corchorus capsularis* L. and *C. olitorius* L.), and kenaf (*Hibiscus cannabinus* L.). They are distinguished into gelatinous (G) or xylan-type, depending on the composition of their secondary cell walls: the former contains high percentages of crystalline cellulose (up to 90% [23]) and low amounts of lignin (e.g., ca. 4% in hemp [24]), while the latter contains xylan and are lignified [25]. Hemp, flax, ramie, and nettle produce G-type bast fibres, while kenaf and jute differentiate xylan-type fibres [23]. The biocomposite industry is interested in the use of natural fibres (e.g., hemp bast fibres) as natural substitutes of man-made fibres: plant fibres are cheaper, renewable, have a low C footprint, and, compared to glass fibres, they raise no health-related concerns [26,27].

We will here focus on textile hemp, as this is a multi-purpose crop which has witnessed a renewed interest [27].

**Figure 1:**
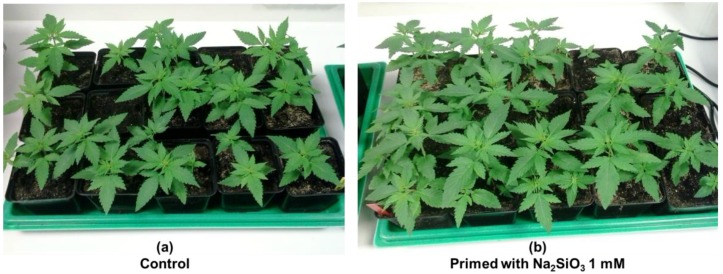
Priming of hemp with Si. (**a**) Non-primed plants; (**b**) Primed plants. The plants are 15 days old.

We have observed a positive effect when hemp seeds are primed overnight with a solution of sodium metasilicate (Na_2_SiO_3_) 1 mM (Figure 1): the germination rate is higher and the plantlets are more vigorous. Experiments in our laboratory are ongoing to assess, from a molecular point of view, the relationship between Si application and bast fibre differentiation. The increased germination and vigor of the hemp plantlets suggests an effect on both primary and secondary growth, processes which can be studied by focusing on the hypocotyl. In this respect, we recently showed the suitability of the hemp hypocotyl for molecular studies focusing on secondary growth and bast fibre formation [28]: the hypocotyl undergoes elongation during the first nine days, then elongation ceases and secondary growth starts. However, gene expression analysis on a set of cell wall-related genes (secondary cellulose synthases *CesA4*-*7*-*8*, an expansin gene *EXPA8*, fasciclin-like arabinogalactan proteins *FLA1*-*3*-*6*-*8*-*10*, phenylpropanoid pathway-related genes *4CL*, *CAD*, *PAL*) did not show any statistically significant changes in expression in the hypocotyl. This finding confirms the latent prophylactic effect of Si on plants unless an exogenous constraint is present (as recently reviewed, [29]). The targeted gene expression analysis, however, does not capture the dynamism of the whole transcriptome; hence, the transcriptomic analysis of hemp hypocotyls will eventually provide more information on the changes in expression after Si supplementation.

**Figure 2:**
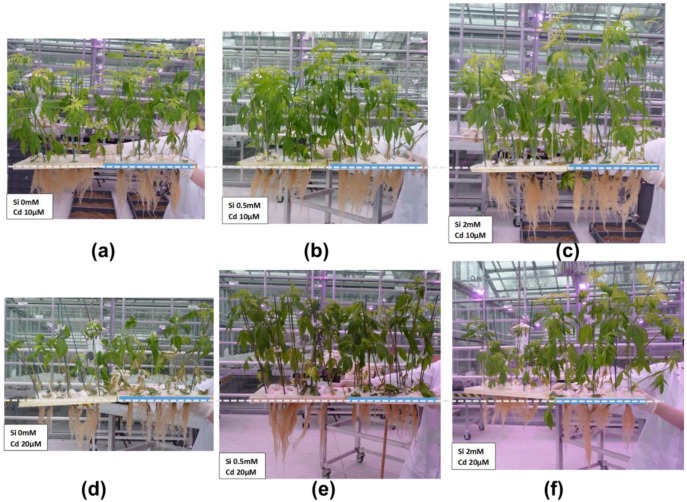
Protective effect of Si against Cd in hemp. (**a**) Plants treated with Cd 10 µM; (**b**) Plants treated with silicic acid 0.5 mM and Cd 10 µM; (**c**) Plants treated with silicic acid 2 mM and Cd 10 µM; (**d**) Plants treated with Cd 20 µM; (**e**) Plants treated with silicic acid 0.5 mM and Cd 20 µM; (**f**) Plants treated with silicic acid 2 mM and Cd 20 µM. The plants were grown in hydroponic culture and are 35 days old.

**Figure 3:**
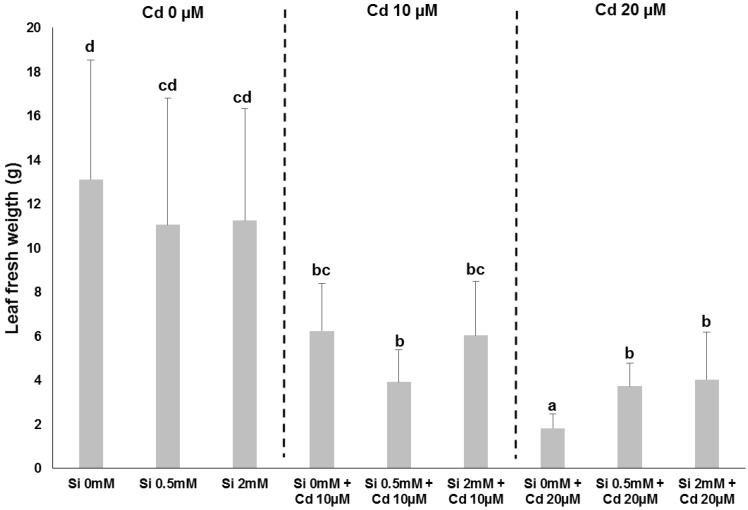
Leaf fresh weight measured on the plants shown in Figure 2. Different letters indicate statistically different values according to the one-way ANOVA test (*p* < 0.05).

The presence of heavy metals in soils is a factor negatively affecting the productivity of crops: for example, in flax Cd was shown to inhibit the growth of seedlings [30] and to impact the cell wall of bast fibres by modifying the assembly and crystallinity of cellulose microfibrils [31]. Si is known to confer protection to plants against abiotic stresses, by priming the cellular response (as recently reviewed [29,32]). We have indeed observed a protective effect of Si (under the form of silicic acid 0.5 and 2 mM) on hemp plants treated with Cd at different concentrations, i.e., 10 and 20 µM (Figure 2): the leaf fresh weight of plants grown with Cd 20 µM is higher under Si supplementation (Figure 3).

The targeted gene expression profiling on a set of cell wall-related genes (involved in both primary and secondary growth; [28]) and on candidates involved in the general stress response (encoding reactive oxygen species-detoxifying enzymes) is ongoing to decipher the molecular response of hemp to Cd, Zn, and Cd/Zn stress under Si supplementation.

## 3. How Can Si Affect Bast Fibre Growth and Development?

The increased vigor and biomass under Si supplementation in hemp (Figure 1 and Figure 2) suggests an effect on the molecular processes regulating both primary and secondary growth. The production of plant biomass is a process relying on the coordination of different events (e.g., transition from elongation to thickening of stem tissues, synthesis of secondary cell walls impregnated with lignin), many of which are regulated by phytohormones. We therefore propose that the stimulatory effects on biomass production under Si supplementation can be (partly) explained by the Si-mediated action on phytohormone biosynthesis. There is paucity (if not a total lack) of data concerning the effects that Si has on the growth and differentiation of bast fibres; in this paragraph, we will propose some hypotheses on the positive effect of Si on bast fibre development and production.

In hemp there are both primary and secondary bast fibres: the former originate from the procambium, while the latter originate from the cambium [33]. Bast fibres in fibre crops fulfil an important role: they support the phloem tissue by enveloping it in a ring of fibre bundles with thick cellulosic walls providing mechanical strength to the vascular system. A stimulatory effect on (pro)cambial activity can be invoked to explain the increased biomass production of hemp plantlets under Si supplementation, either in the absence or presence of an exogenous stress (Figure 1 and Figure 2). In this respect, it should be noted that plant hormones play important roles in the establishment of (pro)cambial cells, which then differentiate the (primary/secondary) xylem and phloem (reviewed by Guerriero and colleagues [34]): auxin, for example, is crucial in the determination of procambial precursor cells, as well as in secondary growth [35] and cytokinins regulate cambial activity during secondary development [36]. An increased (pro)cambial activity triggered by Si can lead to an increased bast fibre differentiation in hemp stems; in addition to that, Si may affect the phytohormone balance regulating important stages of bast fibre development, notably intrusive growth and cell wall thickening. We recently showed that, in elongating hemp bast fibres, transcripts related to the indole glucosinolate metabolic process were upregulated. We proposed that indole glucosinolate synthesis may be related to the wound hormone jasmonic acid (JA) and may regulate the phase of intrusive growth [37]. Interestingly, a relationship has been reported in the literature between Si and JA: Si primes JA-mediated defense response in rice [11]. Additionally, Si is capable of promoting cytokinin biosynthesis, thereby delaying leaf senescence in thale cress and *Sorghum* [38].

In the bottom of hemp stems, the bast fibres develop the gelatinous thick tertiary cell wall and cease elongation. We showed that an enrichment of transcripts related to abscisic acid (ABA) and gibberellin (GA) metabolism—more specifically a transcript, *GA2OX2*, regulating the conversion to an inactive form and thereby controlling GA homeostasis—was highly expressed at the bottom of hemp stems [37]. In the light of its connection with phytohormones, Si may act on both ABA and GA biosynthesis/homeostasis, as well as determine an increase in fibre production and, ultimately, in biomass yield. It would be interesting to test this hypothesis on hemp plants subjected to a condition negatively affecting biomass production, e.g., an abiotic stress (such as heavy metals).

Primary and secondary bast fibres in hemp derive from the procambium and cambium, respectively. Hence, taking into account the Si-induced germination/growth promotion in hemp (Figure 1), it is reasonable to infer a relationship between Si and phytohormones acting on (pro)cambial activity. A phytohormone-mediated stimulatory effect on growth likely exists in fibre crops and may explain the positive effects of Si on biomass production.

It will be highly relevant in the future to quantify phytohormones in hemp stem tissues after Si application, in the absence/presence of an exogenous trigger (e.g., an abiotic stress): internodes located at different stem heights can be sampled and peeled to understand the effects of Si on the balance of phytohormones along the stem regions corresponding to different stages of bast fibre development.

Another element to consider with respect to the role of Si in bast fibre development is the cell wall-associated effect of biogenic silica. Its incorporation in the cell walls of bast fibres may provide enhanced mechanical properties, which help the cell wall to better withstand the high turgor pressure present during the active elongation phase of the fibres.

It is here necessary to also consider the reported increased cell extension observed after Si supplementation at the base of leaves of plants, e.g., rice and other *Poaceae* [39], as a similar mechanism may occur in bast fibres and affect the final physical properties of the material.

## 4. SiO_2_ and Lignocellulosic Biomass: An Industrial and Agricultural Perspective

The association of SiO_2_ with plant cell walls has important consequences on plant biomass properties. For example, by associating with the cell walls, SiO_2_ confers mechanical protection to tissues, thereby lowering the palatability and digestibility by vertebrates and in vertebrates [40,41]. We describe here three aspects associated with the industrial and agricultural use of SiO_2_-rich biomass: (1) the manufacture of more durable biocomposites; (2) both the enzymatic and thermochemical conversion of lignocellulose to biofuels; and (3) the use of Si-rich biochar.

The presence of SiO_2_ in the lignocellulosic biomass confers important physical properties to the resulting biocomposite. One key example is represented by hemp-derived woody biomass, which contains SiO_2_. The hemp stem shows an internal woody core (also known as shives or hurds; [26,33,34]) which is widely used in the construction sector to manufacture insulating panels; the presence of SiO_2_ in hemp hurds favours the formation of a lightweight material, once in contact with lime binder. The resulting material, known as “hempcrete” is a biocomposite which is not only very light, but also durable, since the presence of SiO_2_ protects it against the attack of chewing insects, termites, fungi, rodents. SiO_2_ is additionally fire-retardant.

The presence of SiO_2_ in lignocellulosic biomass affects industrial enzymatic conversion processes. This explains why rice is not the preferred energy feedstock: rice has a SiO_2_ layer which shields the lignocarbohydrate complex from mechanical and enzymatic processing [42]. A recent paper analysing 42 rice mutants demonstrated that a possible way to use rice as energy feedstock concerns the use of mutants showing increased digestibility and supplemented with Si, to overcome any possible growth defect associated with the mutation [43].

It was shown that SiO_2_ associated with paddy straw hinders microbial action by shielding the cellulose/hemicellulose network [42].

Additionally, SiO_2_ can cause fouling and slagging in thermochemical conversion by reacting with K or Ca present in some ashes and by favouring the formation of alkali silicates with a low melting point [44,45]. Breeding programs aimed at reducing the amount of SiO_2_ are promising tools for a more efficient use of herbaceous feedstock ([45] and the references therein).

Quantitative trait loci (QTL) are linked to Si accumulation [46,47,48]; the study of these QTL can be helpful in devising marker-assisted selection strategies of low SiO_2_ herbaceous feedstock.

The decrease of SiO_2_ content in herbaceous species can also be achieved via the downregulation of Si channels and compensation via lignin deposition, to provide mechanical support to the aerial tissues [44]. In this respect, we believe it is important to mention here the engineering of crops with reduced amounts of lignin (without consequences on plant strength) by modulating the expression of transcription factors (TFs). This approach could be used in conjunction with the selection of low-SiO_2_ bioenergy crops to increase the enzymatic and thermochemical conversions without affecting the plant performance. For example, in rice, the expression of the *A. thaliana* TF SHINE (SHN) induced an increase in cellulose and a decrease in lignin, with no impact on plant strength or performance [49].

Synthetic biology has already shown promising results in *A. thaliana* to enhance the digestibility of secondary cell walls. Lignin deposition was rewired by disconnecting cinnamic acid 4-hydroxylase from the fibre regulatory network and at the same time inserting an artificial positive feedback loop driving cellulose deposition in fibres [50].

Synthetic biology approaches can therefore be devised in Si-accumulators to use them more efficiently as energy feedstock.

## 5. Conclusions

Si is a non-essential element that confers increased vigor and resistance to plants in response to numerous environmental constraints. Its use in agricultural practices could therefore be considered to improve the biomass yield of economically important cultivated plants, such as fibre crops. Our preliminary results indeed show a positive effect on hemp primed with Si, as well as a protective effect against Cd toxicity. However, to date, only a handful of studies are available concerning the impact of Si application on the growth and differentiation of fibre crops. In the future, the use of biophysical tools (e.g., micro particle-induced X ray emission, X-ray absorption near edge structure) for the elemental analysis of accumulated Si should be considered to map its distribution in different tissues, in connection with high-throughput transcriptomics and/or targeted gene expression of key candidates (cell wall-related, for example), as well as the quantification of phytohormones in stem tissues.
